# Platelet-expressed immune checkpoint regulator GITRL in breast cancer

**DOI:** 10.1007/s00262-021-02866-y

**Published:** 2021-02-04

**Authors:** Yanjun Zhou, Jonas S. Heitmann, Kim L. Clar, Korbinian N. Kropp, Martina Hinterleitner, Tobias Engler, André Koch, Andreas D. Hartkopf, Lars Zender, Helmut R. Salih, Stefanie Maurer, Clemens Hinterleitner

**Affiliations:** 1 Clinical Collaboration Unit Translational Immunology, Department of Internal Medicine, German Cancer Consortium (DKTK), University Hospital Tuebingen, Tuebingen, Germany; 2 Cluster of Excellence iFIT (EXC 2180) “Image-Guided and Functionally Instructed Tumor Therapies”, University of Tuebingen, Tuebingen, Germany; 3 Department of Hematology, Medical Oncology and Pneumology, University Medical Center of Mainz, Mainz, Germany; 4 Department of Obstetrics and Gynecology, University Hospital Tuebingen, Tuebingen, Germany; 5 Department of Radiology, Memorial Sloan Kettering Cancer Center, New York, NY USA; 6 Department of Medical Oncology and Pneumology (Internal Medicine VIII), University Hospital Tuebingen, Tuebingen, Germany; 7 German Cancer Research Consortium (DKTK), German Cancer Research Center (DKFZ), Partner Site Tübingen, Heidelberg, Germany

**Keywords:** Breast cancer, Immune checkpoint, Platelets, GITRL

## Abstract

Owing to their key role in several diseases including cancer, activating and inhibitory immune checkpoint molecules are increasingly exploited as targets for immunotherapy. Recently, we demonstrated that platelets, which largely influence tumor progression and immune evasion, functionally express the ligand of the checkpoint molecule GITR. This immunoreceptor modulates effector functions of T cells and NK cells with its function varying dependent on cellular context and activation state. Here, we provide a comparative analysis of platelet-derived GITRL (pGITRL) in breast cancer patients and healthy volunteers. The levels of pGITRL were found to be higher on platelets derived from cancer patients and appeared to be specifically regulated during tumor progression as exemplified by several clinical parameters including tumor stage/grade, the occurrence of metastases and tumor proliferation (Ki67) index. In addition, we report that pGITRL is upregulated during platelet maturation and particularly induced upon exposure to tumor-derived soluble factors. Our data indicate that platelets modulate the GITR/GITRL immune checkpoint in the context of malignant disease and provide a rationale to further study the GITR/GITRL axis for exploitation for immunotherapeutic intervention in cancer patients.

**Supplementary Information:**

The online version of this article (10.1007/s00262-021-02866-y) contains supplementary material, which is available to authorized users.

## Introduction

Despite considerable advances in diagnostics and treatment of breast cancer, disseminated disease is still a major challenge in clinical oncology [1]. A better understanding of the processes that influence tumor progression is thus key to improve therapeutic options and ultimately prognosis of patients. The capacity of tumor cells to evade immunosurveillance, i.e., immune escape, is a hallmark of cancer [2] and is greatly facilitated via inhibitory or activating signals for immune cells. Many of the molecules governing the respective pathways are referred to as immune checkpoints as they are crucial regulators of immune responses and self-tolerance [3]. Among the most prominent and thoroughly studied members are programmed cell death receptor-1 (PD-1) and cytotoxic and T lymphocyte-associated molecule-4 (CTLA-4), and their modulation has meanwhile become a mainstay of oncological treatment [3, 4].

Platelets are components of the myeloid leukocyte lineage. Their pathophysiological role in tumor progression is recognized for many years, as they interact with blood-borne tumor cells forming platelet–tumor cell aggregates. Platelets enhance metastasis via multiple mechanisms including supply of growth factors/chemokines, facilitation of endothelial adhesion and transmigration, supporting the establishment of secondary lesions, induction of an epithelial-to-mesenchymal transition and also by facilitating immune evasion [5–7]. We and others have recently shown that a plethora of immune checkpoint molecules are expressed on platelets including LIGHT, OX40L, MHC class I but also glucocorticoid-induced TNF receptor family-related protein ligand (GITRL, also known as TNFSF18) [8–11]. We further demonstrated that platelet-derived non-malignant MHC class I and platelet-derived GITRL (pGITRL) are transferred to tumor cells upon interaction with platelets, which impedes reactivity of NK cells via their cognate receptors [10, 11]. This is particularly noteworthy in the context of the role of GITR as immune checkpoint and present efforts to develop approaches for its therapeutic modulation [3]. However, the prognostic role of platelet-expressed immune checkpoint molecules in tumor patients in general and GITRL in particular so far is largely unknown. In this prospective observational study, we analyzed pGITRL in breast cancer patients compared to healthy volunteers and found pGITRL to be specifically regulated with regard to stage/grade, metastatic events and tumor proliferation. pGITRL serves as a predictive marker of metastasis. In addition, we report on the induction of pGITRL during platelet maturation and upon exposure to tumor-derived soluble factors. Our results point to a novel mechanism by which platelets influence tumor–immune cell interaction and identify pGITRL as pathophysiologically relevant and prognostic factor in breast cancer.

## Materials and methods

### Reagents

Paraformaldehyde was obtained from Affymetrix (Santa Clara, CA). Anti-GITR, anti-GITRL antibodies and the respective isotype control were from R&D Systems (Minneapolis, MN). CD19-FITC, CD41a-PeCy5, CD41a-PE, PAC-1-FITC, CD61-FITC and CD62P-FITC were from BD Pharmingen (San Diego, CA), CD3-APC/Fire and CD56-PeCy7 were from Biolegend (San Diego, CA). The goat anti-mouse PE conjugate was from Dako (Glostrup, Denmark). Biocoll Separating Solution was purchased from Biochrom AG (Berlin, Germany). VPA was from Sigma-Aldrich (St. Louis, MO). Thrombin Receptor Activator Peptide 6 (TRAP-6), collagen and ADP were purchased from Sigma-Aldrich (St. Louis, MO).

### Cell lines

The tumor cell lines Meg01, MCF-7, MDA-MB 231, MDA-MB 468, Hs578T, BT-474, T47d, BT-549 and SK-BR-3 were from German Collection of Microorganisms and Cell Cultures (Braunschweig, Germany).

### Patients

During 2019–2020, blood samples from 79 breast cancer patients treated at the Department of Obstetrics and Gynecology and the Department of Medical Oncology and Pneumology were included in our prospective study. Written informed consent in accordance with the Helsinki protocol was given in all cases. The patient characteristics in detail are given in Table 1.

**Table 1:** Patient characteristics of the breast cancer cohort

Patient characteristics	Total
	(*n* = 79)
Gender	
Female sex, *n* (%)	78 (98.7)
Age	
Age in years, mean–yr ± SD (range)	60.1 ± 13.5 (27 to 87)
TNM classification, *n* (%)	
Stage	
T0	8 (10.1)
T1	25 (31.6)
T2	28 (35.4)
T3	11 (13.9)
T4	7 (8.9)
Node	
N0	43 (54.4)
N1	23 (29.1)
N2	10 (12.7)
N3	3 (3.8)
Metastasis	
M0	59 (74.7)
M1	20 (25.3)
Localization of primary tumor	
Right	35 (44.3)
Left	44 (55.7)
Tumor size	
Histological grading, *n* (%)	
G1	10 (7.9)
G2	33 (41.8)
G3	35 (44.3)
Unknown	1 (1.3)
ER positive, *n* (%)	57 (72.2)
PR positive, *n* (%)	52 (65.8)
Her2 positive, *n* (%)	48 (60.8)
Treatment, *n* (%)	
Adjuvant chemotherapy	20 (25.3)
Neoadjuvant chemotherapy	20 (25.3)
Adjuvant Endocrine therapy	17 (21.5)
Adjuvant radiation	18 (22.7)

Abbreviations: *n* Number; *T* tumor; *N* lymph node; *M*, metastasis; %, percentage; *yr.*, years; *SD* Standard deviation; *G* grading; *ER* estrogen receptor; *PR* progesterone receptor

### Preparation of PBMC and platelets

Preparation of platelets and PBMC was performed as previously described [8].

### Immunofluorescence

For immunofluorescence staining, platelets and Meg-01 cells were fixed in 4% PFA in PBS (10 min at 4 °C). Platelets and cells were blocked using a BSA blocking solution containing 5% BSA, 0.2% Triton X-100, 0.1% Tween for 60 min. As primary antibody we used anti-GITRL (1:200, R&D Systems) and anti-CD61 (1:500, ThermoFisher, St. Louis, MO); as secondary antibodies Alexa-Fluor 594 labeled anti-rabbit (1:1000, Invitrogen, Carlsbad, CA) and Fluor 488 labeled anti-mouse (1:1000, Invitrogen) were used. Slides were mounted in fluorescent mounting medium; for Meg-01 cells 0.5 μg/ml Hoechst was used for counter-staining. Plasma membranes were stained using Dil (ThermoFisher), nuclear staining was done via NucBlue™ (ThermoFisher) according to manufacturer’s instructions. Pictures were acquired using an Olympus BX63 microscope and a DP80 camera (Olympus, Shinjuku, Japan).

### Platelet aggregation

Platelet aggregation was analyzed here using the 4-channel light transmission platelet aggregometer APACT 4004 (Elitech, Puteaux, France) according to the manufacturer’s instructions. For platelet stimulation ADP (10 µM) and TRAP-6 (10 µM) was used.

### Flow cytometry

Flow cytometry was performed using fluorescence-conjugates or unlabeled antibodies at saturating concentrations followed by a goat anti-mouse PE conjugate (1:100) as secondary antibody. Analysis was performed using a FACS Canto or a FACS Fortessa (BD Biosciences, Heidelberg, Germany). Percent positive cells were calculated as follows: “percent surface expression obtained with specific antibody”—“percent surface expression obtained with isotype control”. B cells were characterized by CD19+, T cells by CD3+, and NK cells by CD56+ CD3 −. Dead cells were excluded using Fixable Aqua (Invitrogen) after extracellular staining according to the manufacturer’s instructions. Platelets were selected CD41a+ and CD62P− (resting) or CD62P + (activated). The LEGENDplex HU Essential Immune Response Panel Standard (Biolegend) was used according to manufacturer’s recommendations.

### Statistics

For continuous variables student’s* t* test, Mann–Whitney *U* test or one-way ANOVA was used. For categorical data, we used chi‐squared test or Fisher's exact test. Correlation of platelet activation and GITRL expression and Ki67 was analyzed using simple linear regression analysis. The predictive value of pGITRL was evaluated by examining the area under the receiver‐operator characteristic (ROC) with a confidence interval of 95%. For correlation studies of pGITRL and different clinical parameters Odds ratios (OR) were calculated. High pGITRL expression was defined as follows: pGITRL high = mean pGITRL (HD) + 2SD pGITRL (HD). All statistical tests were considered significant when *p* was below 0.05.

## Results

### Expression of GITRL on platelets and its counterpart GITR on lymphocytes of breast cancer patients

**Fig. 1:**
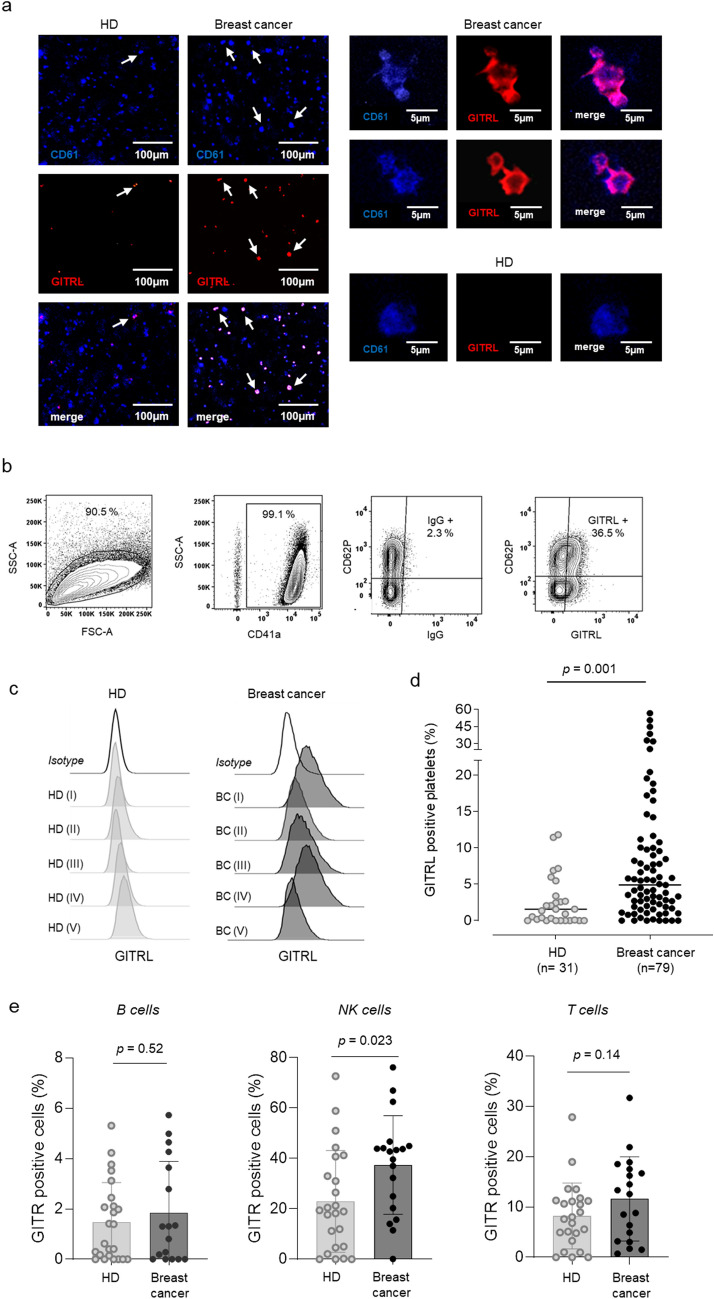
Expression of GITRL by platelets and its receptor GITR on lymphocytes. **a** Immunofluorescence analysis of pGITRL (labeled in red) and CD41 (labeled in blue) expression in platelets from a HD and a breast cancer patient. **b–d** GITRL surface levels of platelets from a breast cancer patient and HD were investigated by flow cytometry. **b–c** Gating strategy used to analyze platelets ex vivo (**b**) and representative results obtained from the patient and HD are shown (**c**). **e** GITR surface levels on PBMC subpopulations from breast cancer patients and HD are shown. (**a, b**) Representative data of one experiment from a total of at least three with similar results are shown

Platelets are the first encounter circulating tumor cells make when entering the blood stream, and this facilitates various steps of the metastatic cascade [6]. While platelets of healthy individuals are known to express moderate levels of the immune checkpoint molecule pGITRL [10], we here investigated the expression of pGITRL on platelets from breast cancer patients. The clinical characteristics of the 79 breast cancer patients included in our study are given in Table 1. In a first step we performed immunofluorescence analysis of platelets from healthy donors and breast cancer patients. Platelets were identified by the platelet integrin β3 (also referred to as GPIIIa or CD61). The pGITRL levels detected in platelets from breast cancer patients were significantly higher as compared to healthy donors (HD). According to our immunofluorescence analysis, pGITRL was localized predominantly membranous (Fig. 1a).

Next, we utilized flow cytometry to confirm that pGITRL is displayed on the surface of platelets ex vivo. Platelets were defined as CD41a-positive subcellular fragments and GITRL levels were analyzed with regard to the platelet activation marker P-selectin (CD62P) (Fig. 1b). pGITRL levels in patients revealed a substantial inter-individual variability but were overall significantly higher in the analyzed 79 breast cancer patients compared to our cohort of 31 HD (*p* = 0.001) (Fig. 1c–d).

We then comparatively analyzed the expression of the cognate receptor GITR on lymphocyte populations among the PBMC of the breast cancer patients compared to the HD (Fig. 1e, Suppl. Figures 1, 2, 3). Whereas GTIR expression levels were slightly decreased in CD19 positive B-cells (*p* = 0.29), we detected significantly increased GITR expression on NK cells of patients (*p* = 0.036). Analysis of T cells revealed a tendency towards higher expression levels that closely failed to reach statistical significance (*p* = 0.064) (Fig. 1e). Notably, lymphocyte activation marker, particularly CD69 were upregulated in B, NK and T cells of breast cancer patients, pointing to an ongoing immune response in this context (Supplementary Fig. 2a–f). Moreover, low levels of pGITRL were associated with higher NK cell activation compared to levels of pGITRL, confirming previous data that pGITRL acts as NK inhibitory ligand. In contrast, patients with high levels of pGITRL tended to higher CD69 expression in T cells, alike supporting existing data on the activating function of GITR in T cells (Supplementary Fig. 3c–d).

**Fig. 2:**
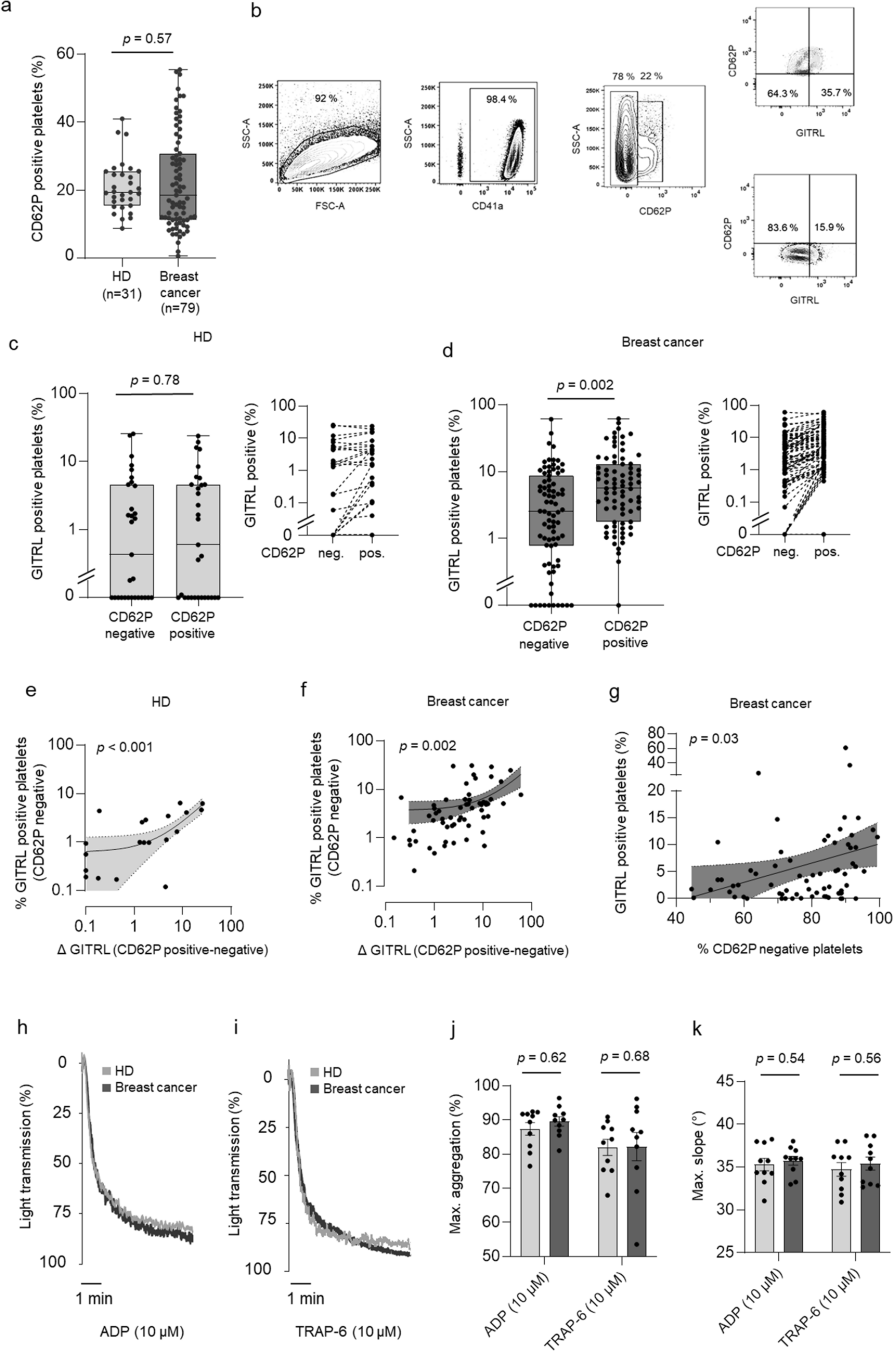
Correlation of pGITRL expression and platelet activation. **a**–**g** Expression of pGITRL and the platelet activation marker P-selectin (CD62P) in platelets from HD and breast cancer patients ex vivo. **b** Gating strategy used to analyze pGITRL levels with regard to platelet activation*.* **c–d** pGITRL positivity with regard to CD62P expression in HD (**c**) and breast cancer patients (**d**) is shown. **e–f** Correlation of pGITRL expression in CD62P negative platelets and GITRL expression change (Δ GITRL, defined as “%GITRL expression in endogenously activated, CD62P positive platelets”—“%GITRL expression in CD62P negative platelets”) in HD (**e**) and breast cancer patients (**f**). **g** Correlation of pGITRL expression in resting platelets in breast cancer patients and percentages of CD62P negative platelets. **h–k** Platelet aggregation was studied in the presence or absence of classical platelet agonists ADP or TRAP-6. Percentage of light transmission during the indicated time interval (**h**, **i**) and maximum aggregation levels or gradient (**j**, **k**) are shown (HD, light grey, breast cancer patients, dark grey; *n* = 10 each)

**Fig. 3:**
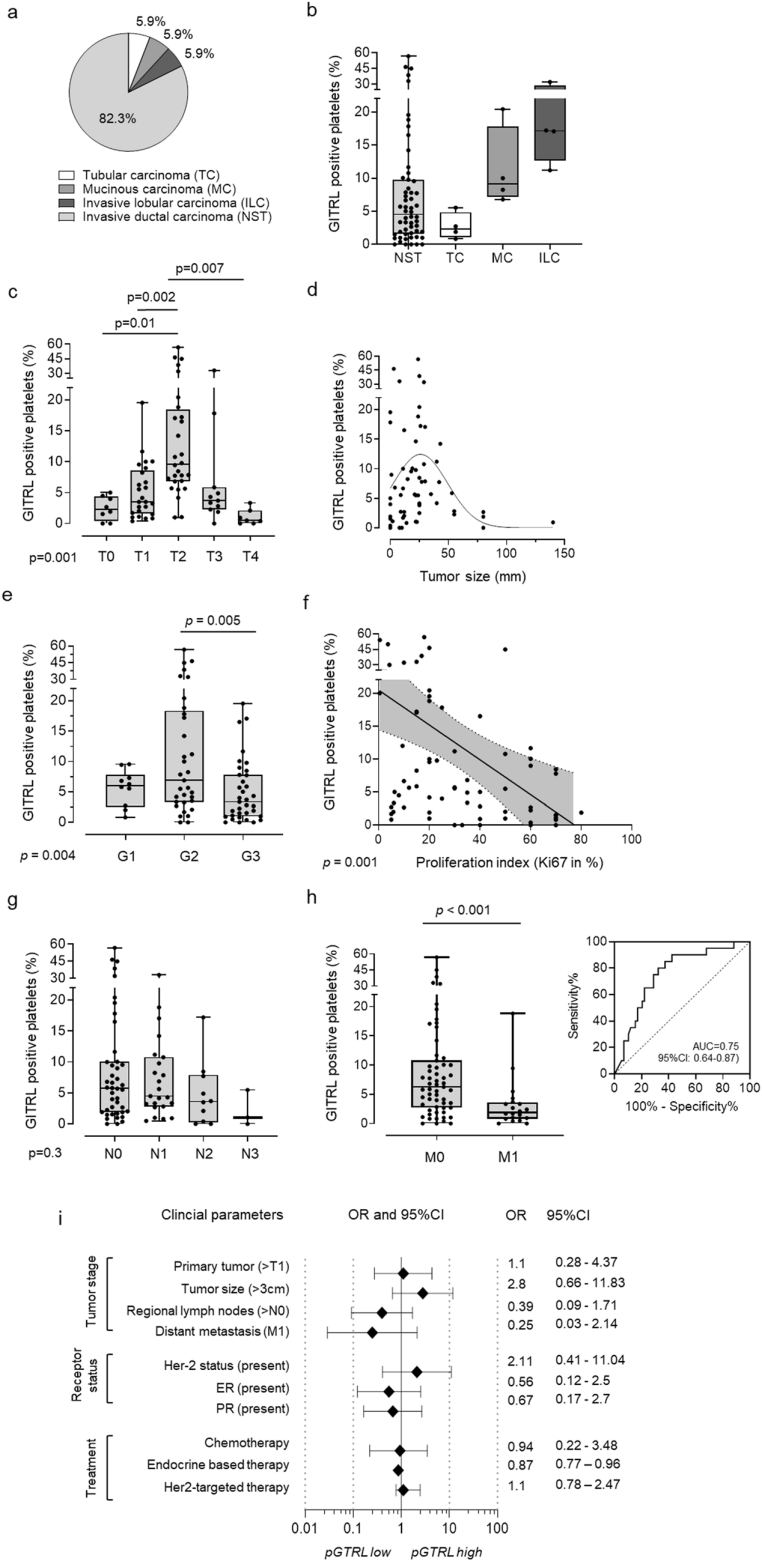
Association of pGITRL with clinical parameters of breast cancer patients. **a, b** Classification of breast cancer patients used in this study according to histopathological subtype (**a**) and association with pGITRL levels (**b**).** c**–**e** Expression of pGITRL according to the breast cancer tumor stages (T) (**c**), tumor size (**d**) and different tumor grades (G1–3) (**e**).** f** Correlation of pGITRL expression and tumor proliferation (% Ki67 positive tumor cells).** g**,** h** Expression of pGITRL according to the breast cancer lymph node invasion (N) (**g**) or occurrence of metastasis (M) (**h**). The predictive value of pGITRL for metastasis was analyzed using ROC. (**i**) OR of several clinical parameters and their association with pGITRL expression are shown. pGITRL high was defined as follows: mean pGITRL (HD) + 2SD pGITRL (HD)

### Correlation of pGITRL expression and platelet activation

As described previously, pGITRL can be upregulated in vitro by classical platelet agonists including collagen, thrombin and ADP [8, 10]. However, little is known about the association of pGITRL expression and platelet activation in larger cohorts of patients. We therefore assessed the association of pGITRL levels and platelet activation state in the 31 HD and 79 breast cancer patients.

As a first step, we determined the preexisting activation (CD62P expression) of platelets ex vivo in our cohort, which revealed 19.4% CD62P-positive platelets (95%CI: 10.4–38.6) in HD and 18.6% (95%CI: 6.3–49.8) in breast cancer patients and thus similar platelet activation levels (*p* = 0.57, Fig. 2a). This was also observed when employing GpIIb/IIIa (PAC-1) as platelet activation marker (Supplementary Fig. 5). While the endogenous levels of CD62P and PAC-1 were comparable in both, HD and breast cancer patients, platelet activation led to a more effective upregulation of CD62P as compared to PAC-1 likewise in HD and patients. Thus, we performed the following analyses using CD62P which appeared to represent platelet activation state in our setting more sensitively.

In a second step, we studied the pGITRL level with regard to CD62P expression (Fig. 2b). No relevant differences in pGITRL in the activated (CD62P-positive) and resting (CD62P-negative) platelet fraction was observed in HD (Fig. 2c). In contrast, in breast cancer patients pGITRL was significantly enhanced in the CD62P-positive platelet fraction (*p* = 0.002, Fig. 2d). Of note, the basal pGITRL level of resting (CD62P-negative) platelets was significantly associated with the extent of GITRL upregulation upon platelet activation (Δ GITRL) in platelets from both, HD and breast cancer patients (Fig. 2e–f). Interestingly, pGITRL expression in resting platelets from breast cancer patients correlated with the percentage of resting (CD62P-negative) platelets, indicating that patients exhibiting a large fraction of resting platelets tend to express higher amounts of pGITRL (*p* = 0.03, Fig. 2g).

To further evaluate platelet activity with regard to their function, we performed platelet aggregation studies in vitro in the presence or absence of the classical platelet agonists ADP (10 µM) and TRAP-6 (10 µM), respectively (Fig. 2h–k). Substantial rates of maximal platelet aggregation and the maximal gradient of aggregation could be observed under shear stress in both groups. However, no significant differences were observed between patients and HD (Fig. 2j–k). However, platelets with increased levels of pGITRL tended to higher aggregation in both HD and breast cancer patients (Suppl. Figure 4).

**Fig. 4:**
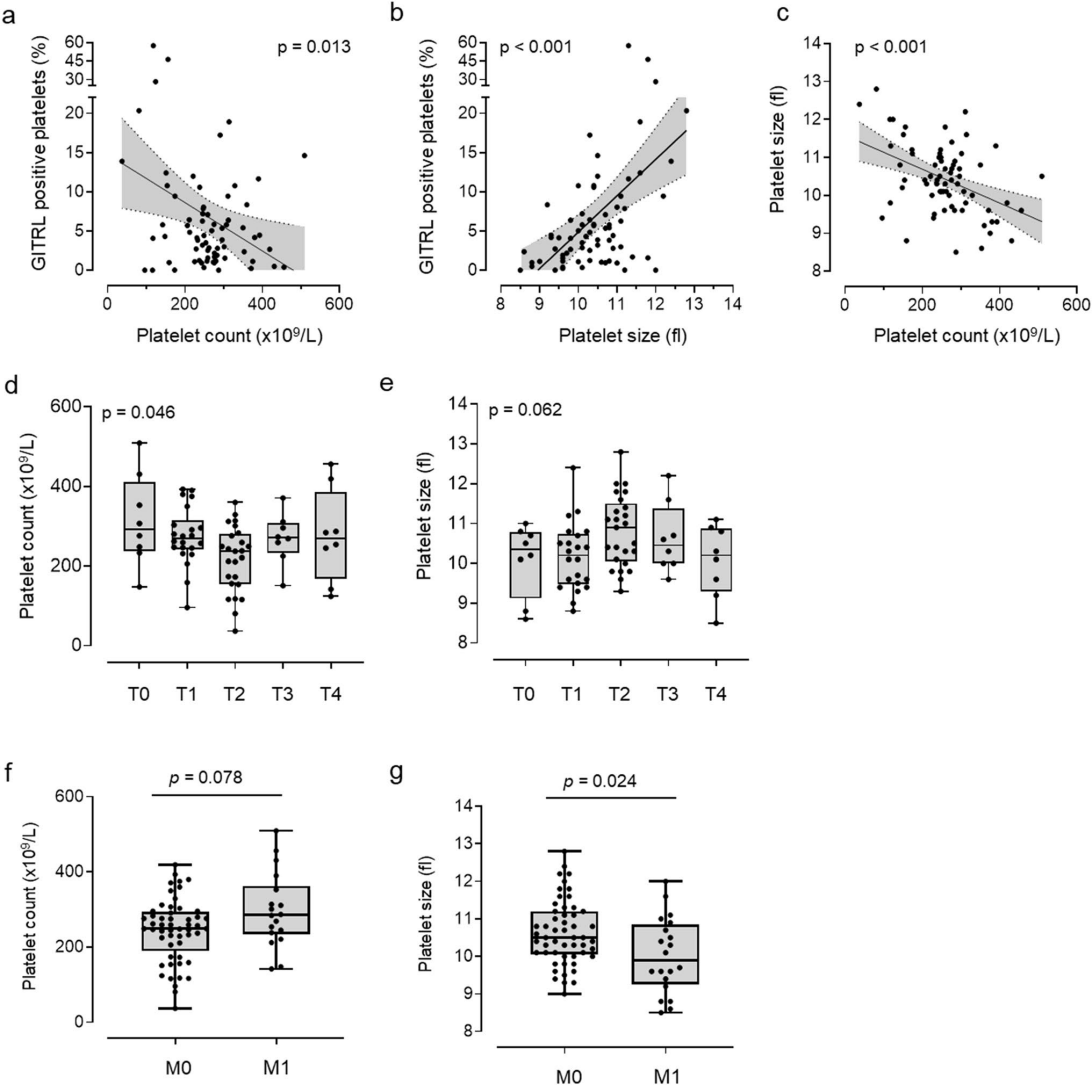
Association of platelet count/size with pGITRL level and tumor stage.** a**–**b** Correlation of platelet count and platelet size with pGITRL expression. **c** Correlation of platelet count and size.** d**–**e** Correlation of platelet count, platelet size and tumor stage (T0–T4) and (**f**–**g**) occurrence of metastasis (M0, M1) in 71 breast cancer patients

### Association of pGITRL expression with clinical parameters in breast cancer

As the observed increase in expression of GITRL on platelets from breast cancer patients suggests an involvement in pathophysiology, we studied the relationship between pGITRL expression and clinical parameters in our patients. Our study included cases with tubular carcinoma (TC), mucinous carcinoma (MC), invasive lobular carcinoma (ILC) and invasive ductal carcinoma (NST), while NST was most represented. Median pGITRL level were low in TC and MC patients, whereas intermediate levels were found in MC and highest in ILC.

We also found that pGITRL is highly regulated among different tumor stages T0–T4. Interestingly, pGITRL levels where significantly higher in the intermediate (T2) stage compared to T0 (*p* = 0.01) or T1 (*p* = 0.002) stages, while T4 tumor patients displayed lowest pGITRL levels (Fig. 3c). Of note, our finding that pGITRL were highest at intermediate T stages of the tumor could be recapitulated with regard to histological grades G1–G3, where most pronounced pGITRL expression was observed in patients with G2 tumors. Alike in cases of patients with T4 stage tumors, patients with histological high-grade G3 tumors displayed lower levels of pGITRL as compared to G2 tumors (*p* = 0.005) (Fig. 3e). Analyses of the proliferation index (Ki67 expression) of tumors which identifies as prognostic marker of bad outcome negatively correlated with pGITRL levels in the respective patients (*p* = 0.001) (Fig. 3f). This is congruent with our findings regarding the tumor T stages and histological G grading (G1–G3).

In contrast, there was no significant association between pGITRL expression and lymph node invasion of the tumor (N0–N3) in the respective patients (Fig. 3g). However, pGITRL levels were negatively associated with the occurrence of metastasis (*p* < 0.001 (Fig. 3h), which is in line with our observation that platelets from advanced tumor stages (T4) displayed low pGITRL surface levels. Since dissemination of tumor cells negatively correlates with survival in breast cancer, we further evaluated the predictive value of pGITRL levels using receiver-operating characteristic (ROC) analysis. Remarkably, with an AUC of 0.74 (95%CI: 0.61–0.87), a specificity of 97.3% (95%CI: 85.8–98.5%) and a positive predictive value (PPV) of 90% (95%CI:54.9–98.5%) pGITRL was found to be a predictive marker in our patient cohort (Fig. 3h). To consider the role of pGITRL expression in the larger clinical context, we calculated the Odds ratios (OR) for multiple clinical endpoints (Fig. 3i). Interestingly, tumor sizes > 3 cm were associated with higher pGITRL levels (OR 2.8) whereas a regional lymph node invasion (N > 0) (OR 0.39) and distant metastasis (OR 0.25) were more likely associated with low pGITRL expression. Regarding to the histopathological expression of Her2 we observed higher level of pGITRL in Her2 positive breast cancer patients (OR 2.11). ER (OR 0.56) and PR (0.67) was positively associated with lower pGITRL. Of note, pGITRL levels appear to be independent of different treatment modalities applied within this cohort.

### Correlation of platelet count/size with pGITRL level and tumor stage

Next, we set out to determine whether clinically relevant platelet characteristics, i.e., platelet count and size, would associate with pGITRL levels, tumor stage or the occurrence of metastasis in our breast cancer cohort. pGITRL expression was particularly enhanced in patients with low platelet count, but enhanced platelet size (Fig. 4a–b). However, platelet count in our breast cancer cohort correlated inversely with platelet size (Fig. 4 c). In line with the aforementioned observation that pGITRL expression was most pronounced in intermediate tumor stages (especially T2), platelet count appeared to be lowest in these patients while platelet size tended to be highest in the same groups (Fig. 4d–e). Similarly, pGITRL levels were high in non–metastasized patients (M0) and given its inverse relationship with platelet count, non-metastasized patients (M0) per se displayed lower platelet count (Fig. 4f). Of note, platelet size was higher in this group (Fig. 4g).

### Regulation of platelet-precursor expressed GITRL

**Fig. 5:**
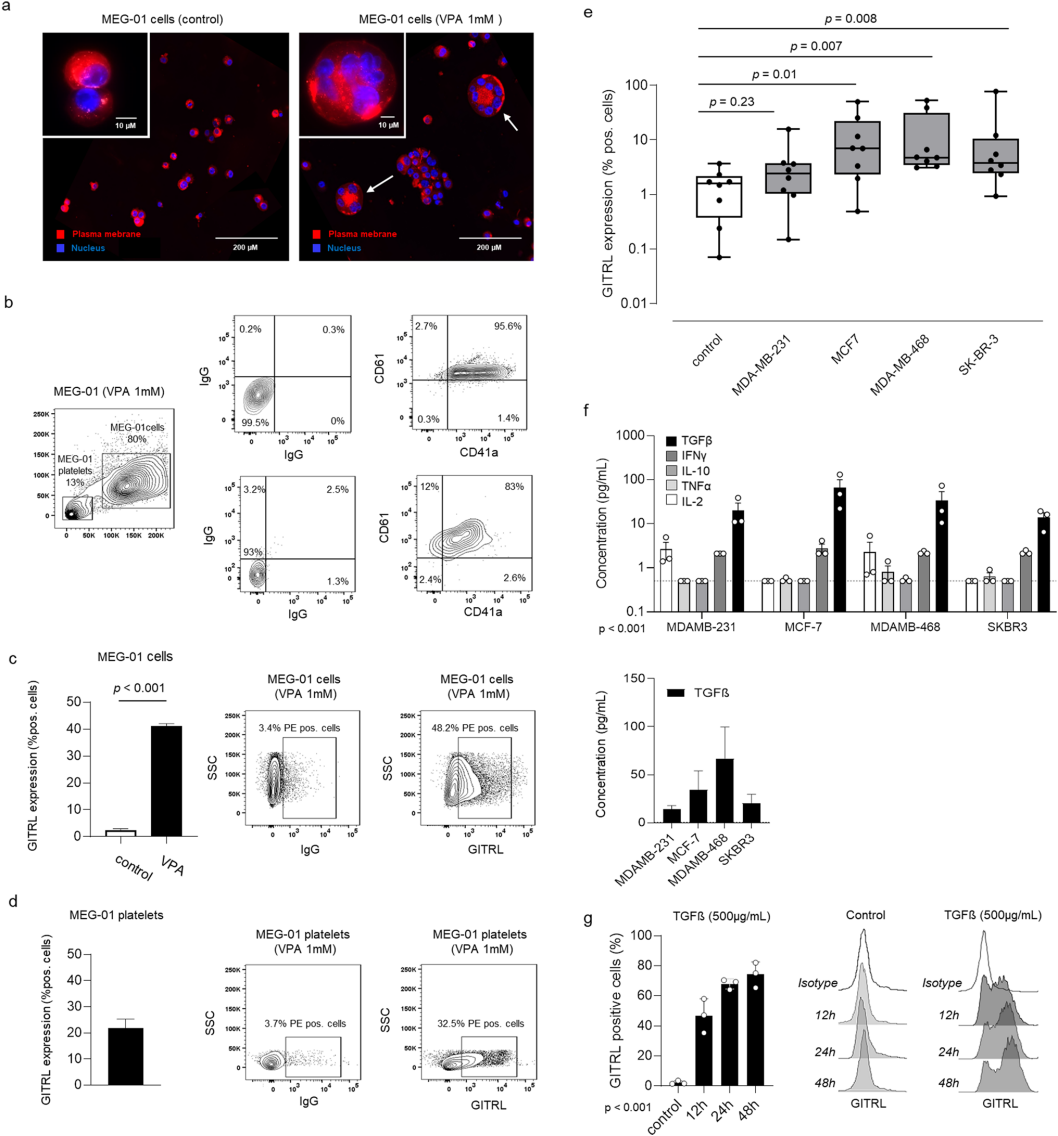
Regulation of platelet-precursor expressed GITRL.** a** Immunofluorescence analysis of MEG-01 cells treated with VPA for 14 days. The plasma membrane was stained using Dil®. Nuclei were counterstained with NucBlue™.** b** Surface levels of thrombopoietic marker on MEG-01 cells and MEG-01 platelets were investigated by flow cytometry. **c**–**d** Surface expression of GITRL on MEG-01 cells and MEG-01 derived platelets treated with VPA (1 mM). The gating strategy displays representative data of one experiment from a total of at least three with similar results. **e** GITRL expression on MEG-01 cells after co-cultivation with conditioned medium of MDA-MB-321, MCF-7, MDA-MB-468 and SK-BR-3 cells for 24 h *n* = 5. **f** Conditioned medium of breast cancer cell lines has been generated as in (e) and subsequently the indicated cytokines were measured by LEGENDplex. Dotted lines indicate the detection limit of the assay. *n* = 3 **g** GITRL expression on MEG-01 cells which were cultured in the presence or absence of TGFβ for 12, 24 or 48 h

Since pGITRL was found to associate with tumor progression in breast cancer, we analyzed whether and how GITRL might be regulated during platelet development and whether the disease impacts pGITRL levels. As an in vitro model for megakaryopoiesis, we induced maturation of the megakaryoblastic MEG-01 cells to a megakaryocytic phenotype using VPA [12]. Immunofluorescence analysis confirmed the generation of large, multinuclear cells over 14 days of culture, which served as a model for megakaryocytes (Fig. 5a–b). We then analyzed expression of GITRL on megakaryoblastic (absence of VPA) or megakaryocytic (presence of VPA) MEG-01 cells and the platelets generated by the latter. Interestingly, we observed a relevant upregulation of GITRL expression during MEG-01 maturation in megakaryocytic MEG-01 cells (Fig. 5c) as well as a relevant GITRL expression on MEG-01 derived platelets (Fig. 5d). We next evaluated whether breast cancer cells can influence pGITRL expression during megakaryopoiesis. To this end, we seeded breast cancer cells and harvested the resulting supernatant, i.e., conditioned medium, which contains soluble tumor-derived factors and cultured MEG-01 cells in the presence or absence of these soluble tumor cell-derived factors for 24 h. Subsequently pGITRL expression was assessed by flow cytometry. Whereas supernatant of MDA-MB-231 cells did not alter GITRL expression, soluble factors derived from MCF-7, MDA-MB-468 and SK-BR-3 cells led to a significant upregulation of GITRL on MEG-01 cells (Fig. 5e). To assess which factors present in the conditioned medium may regulate GITRL expression, we determined release of a panel of tumor-associated cytokines, namely TGFβ, IFNγ, IL-10, TNF and IL-2. Of all tested cytokines, TGFβ was the only one present at elevated levels (> 10 pg/mL). Interestingly, enhanced levels of TGFβ were found in the conditioned media of MCF-7 and MDA-MB-468 cells which induced GITRL expression in MEG-01 cells (Fig. 5f). To confirm our hypothesis, that in fact TGFβ regulates GITRL expression in our model, we cultured MEG-01 cells in the presence or absence of recombinant TGFβ. The latter clearly led to elevated GITRL positivity in MEG-01 cells in vitro.

Summarizing, we here show that expression of GITRL is not only induced during megakaryopoiesis but can be substantially enhanced by tumor-derived soluble factors.

## Discussion

GITR is a member of the TNFR superfamily. Its cytoplasmic domain shows substantial homology with other costimulatory TNFR family members like 4-1BB, OX40 or CD40 [13]. While also being present in certain tissues, GITR is mainly expressed on immune cells, most prominently on T and NK cells [14]. Its ligand GITRL is expressed on dendritic cells, macrophages, B cells, endothelial cells but also on tumor cells of various origins [15, 16] and tumor-derived GITRL affects immune responses [16, 17]. Interestingly, we recently reported that GITRL is expressed by platelets and thereby negatively modulates NK cell antitumor reactivity [10]. Given the prominent role of immunosurveillance in cancer [2], we here study the association of the immunomodulatory molecule pGITRL with tumor progression with a focus on breast cancer.

We demonstrate for the first time, that pGITRL expression is significantly enhanced in breast cancer patients compared to healthy individuals. With regard to its cognate receptor, CD19 + B cells of breast cancer patients displayed a trend towards lower GITR expression, whereas NK cells showed higher expression levels compared to HD, an observation which in T cells slightly failed to reach statistical significance. This is in line with reports on an increased fraction of GITR-expressing T-cell subsets in tumor-positive lymph nodes from patients with advanced breast cancer [18]. In our analyses GITR expression on NK cells was not correlated with pGITRL levels whereas GITR expression on T cell was negatively associated with pGITRL. Albeit, NK activation was inversely correlated with pGITRL expression, supporting our previous data on the NK-inhibitory propensities of pGITRL. T cell activation however was directly dependent on pGITRL level, suggesting an activating function of GITR in T cells.

The relevance of these findings needs to be further investigated in a well-powered patient cohort, as it may be indicative of a crosstalk between platelet-coated tumor cells and lymphocytes via the GITR/GITRL axis.

As platelets upregulate GITRL following activation which occurs upon encounter of malignant cells entering the bloodstream [10], we next studied the expression of pGITRL with regard to platelet activation state in breast cancer patients. Interestingly, the fraction of activated platelets (CD62P+ or PAC-1+) did not significantly differ between tumor patients and healthy controls, although platelets derived from tumor patients showed a higher inter-individual variability with regard to their P-selectin (CD62P) expression compared to healthy donors. This might be due to a hyperactive state which can be associated with a malignant platelet phenotype [19, 20]. Albeit, it should be considered that the molecules associated with platelet activation are differently regulated and thus most certainly have different kinetics also with regard to disease state and their putative encounter with circulating tumor cells. Notably, based on so far available data, it remains unclear how many platelets would be activated by the very few metastasizing cells entering the blood stream and whether this fraction would be detectable. We also studied platelet function ex vivo and obtained similar aggregation levels in platelets from both patients and HD. This setup does however not account for factors present in the tumor microenvironment which may induce altered sensitivity of platelets [21]. Beyond that, parameters like tumor stage certainly contribute to the same as patients with late-stage metastatic tumors were found to have higher platelet reactivity [22]. Interestingly, platelets with increased levels of pGITRL tended to more pronounced aggregation in presence of the classical agonists ADP or TRAP-6. These data are in line with the finding that pGITRL expression was positively correlated with the fraction of CD62P negative platelets and supports the hypothesis of a hyperactivity state in the subpopulation of GITRL positive platelets. However, further studies are certainly needed to elucidate the regulation of platelet (hyper-)sensitivity especially in this particular context. Higher pGITRL expression was observed on activated platelets of tumor patients, which may be the result of (i) reprogrammed megakaryopoiesis in cancer patients [23], (ii) protein synthesis in platelets [24–26] and/or (iii) preformation/storage of pGITRL in platelet granules—which appears to be more plausible than de novo protein synthesis. This is even more since we recently demonstrated that GITRL is found in the cytoplasm of megakaryocytes and that pGITRL is rapidly translocated to the platelet surface upon activation [10]. The same has also been reported for other platelet-expressed immune checkpoint molecules including CD40L and TWEAK [27, 28]. Additional support for our hypothesis is derived from the observation that the extent of GITRL upregulation upon platelet activation (Δ GITRL) is correlated with the basal GITRL expression on resting platelets. Together with our findings that patients with a large fraction of resting platelets overall express higher levels of GITRL, this might also point to an increased sensitivity of platelets towards activating stimuli.

To further evaluate the role of pGITRL levels in breast cancer, we also studied whether its expression is associated with clinical characteristics. Our observation, that patients with intermediate tumor stages (T2) and the tendency that histopathological subtypes (MC, ILC) showed the highest pGITRL expression levels appears surprising at first. A strict correlation of tumor size and pGITRL expression was not observed since patients with advanced tumor stages (T3–4) showed decreased pGITRL level. These finding however, is in line with the fact that patients with metastatic disease, higher tumor grading (G3) and higher proliferation rates (Ki67 index) also displayed lower pGITRL levels. Together these data indicate that pGITRL expression may be specifically regulated among different tumor stages which suggests a role of platelets and pGITRL in orchestrating the complex immunomodulation during progression of solid tumors. It was observed that platelet count in our study was inversely associated with pGITRL levels and positively correlated with platelet size. Moreover, platelet count was inversely correlated with platelet volume in breast cancer patients. The observation that pGITRL was negatively associated with platelet count might indicate an increased subpopulation of regenerated platelets after thrombocytopenia. Since the initial hypothesis that platelet size reflects platelet age could not hold up, the finding that pGITRL was positively correlated with platelet volume might reflect the fact that reprogrammed megakaryopoiesis which occurs in the context of malignant tumors [23] lead to production of qualitatively altered platelets (tumor-educated platelets, TEP) with increased platelet volume [29]. Beyond that, an important parameter will ultimately be the fact whether platelets are indeed tumor-infiltrating which is thought to be associated with a bad prognosis but was not assessed in our study [30].

Of note, differential expression of the cognate receptor GITR has been reported in several infectious/inflammatory illnesses depending on the state of the disease [31]. Notably, GITR signaling activates T effector cells while it inhibits NK cells [3, 16]. Our data may indicate that pGITRL expression negatively correlates with tumor progression. This is counterintuitive at first glance, but could suggest that inhibition of NK cells by high expression of pGITRL is particularly beneficial at intermediate tumor stages. However, in advanced tumor stages, when cancer cells engage T cell checkpoints including PD-1 and CTLA4 [32], stimulation of GITR might be disadvantageous for the tumor, as stimulation of GITR also inhibits the suppressive properties on regulatory T cells [33]. The latter express high levels of GITR and their suppressive properties are inhibited via GITR signaling. Thus, low pGITRL expression may be associated with enhanced regulatory T cell activity. Since regulatory T cells are known to impair both effector T and NK cells, a pGITRL low platelet phenotype might be selected during cancer progression in a complex immunoediting process [34].

Interestingly we observed GITRL expression during maturation leading to GITRL positive subcellular CD41 positive “platelet-like” particles. This might indicate that GITRL on platelets is regulated during megakaryopoiesis. The regulation of GITRL is only partially understood in cancer and inflammation and innate immune signaling such as Toll like receptor activation contribute to its complexity [33, 35]. We investigated the potential effects of breast cancer-derived factors on thrombopoietic GITRL expression using the MEG-01 model and found that soluble factors released by breast cancer cells induced GITRL expression. A manifold of cytokines including IL-2, IL-6, IL-8 or IFN-γ have been described to be secreted by breast cancer cells [36, 37]. The conditioned media used in our study contained large amounts of TGFβ among all tested cytokines. The level of TGFβ present appeared to associate with the extent of GITRL induction by the same supernatant, suggesting that TGFβ may—at least in part—be responsible for regulation of GITRL in this setting. In line, addition of recombinant TGFβ could recapitulate these effects. Our results extend available data as Ni and colleagues report on the induction of GITRL on dendritic cells by TGFβ which is present in the tumor microenvironment [38].

Several clinical trials investigate GITR as target in various malignant entities (NCT01239134, NCT02598960). Moreover, checkpoint therapy using anti-PD-1 and anti-GITR antibodies might be particularly useful in combination therapies [39]. Beyond providing further insight in the possible role of platelet-expressed checkpoint molecule pGITRL in the immune privilege and pathophysiology of solid tumors, our work identified pGITRL as a novel prognostic and predictive biomarker for breast cancer. Implementing further information on the phenotypic imprint of platelet-expressed immune checkpoint molecules [8] which is part of ongoing studies will certainly further support our understanding of platelets as valuable diagnostic tools. Platelets have already been suggested as biomarkers as they are readily available for liquid biopsies [40, 41]. Especially in breast cancer, as shown in our study, pGITRL might be useful as a marker of metastasis.

### Supplementary Information

Below is the link to the electronic supplementary material.

Supplementary file 1 (PDF 1.15 Mb)

## Abbreviations

AUC: Area under the curve
CTLA-4: Cytotoxic T lymphocyte-associated molecule-4
GITR: Glucocorticoid-induced TNF receptor family-related protein
GITRL: Glucocorticoid-induced TNF receptor family-related protein ligand
HD: Healthy donor
OR: Odds ratio
PBMC: Peripheral blood mononuclear cells
PD-1: Programmed cell death receptor-1
PD-L1: Programmed cell death ligand-1
pGITRL: Platelet-derived glucocorticoid-induced TNF receptor family-related protein ligand
PPV: Positive predictive value
ROC: Receiver‐operator characteristic
SD: Standard deviation
TNF: Tumor necrosis factor
TNFR: Tumor necrosis factor receptor
