# Supplementary material for: Platelet-expressed immune checkpoint regulator GITRL in breast cancer
Source: Cancer Immunol Immunother. 2021 Feb 4;70(9):2483–96. doi: 10.1007/s00262-021-02866-y (PMC8360840; doi:10.1007/s00262-021-02866-y)
Supplement: Supplementary file 1 — (PDF 1.15 Mb) [file 262_2021_2866_MOESM1_ESM.pdf]

Supplementary Figures and Informations

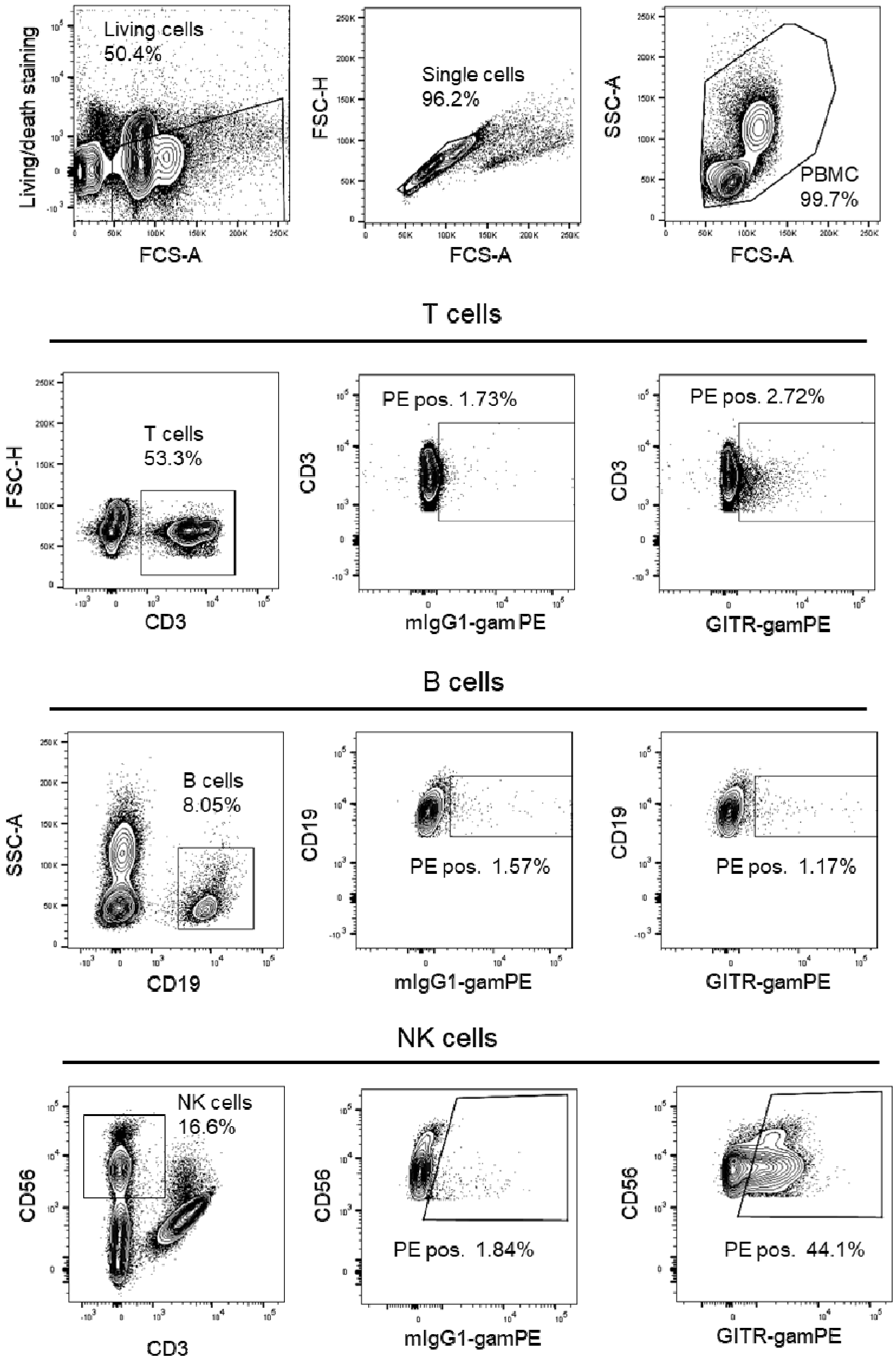

Suppl. Figure 1: Gating strategy to analyze GITR on B,T and NK cells

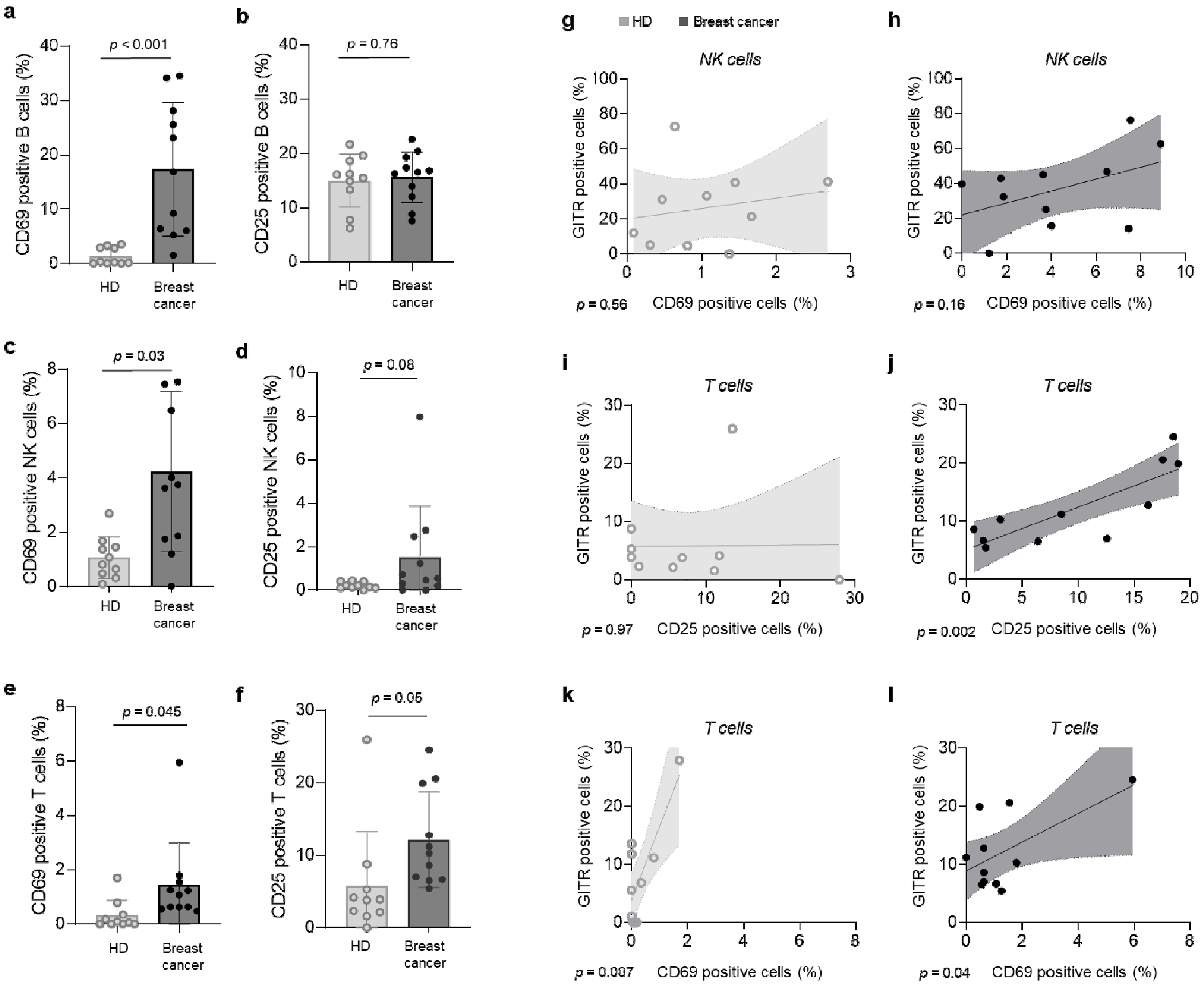

Suppl. Figure 2: Immune-characterization of healthy donors and breast cancer patients. (a) The expression of CD69 in B cells in breast cancer patients (n=11) is significantly increased compared to healthy donors (HD) (n=10). **(b)** However, no differences regarding CD25 expression in B cells was observed. **(c)** Breast cancer patients displayed significantly higher levels of CD69 positive NK cells compared to healthy donors (HDs). **(d)** CD25 positive NK cells tended to be increased in breast cancer. **(e-f)** Compared to HD, breast cancer patients showed higher levels of activated (CD69 positive or CD25 positive) T cells. This data suggest a high proportion of activated immune cells (B, NK and T cells) in breast cancer patients. **(g-h)** In contrast to HDs, the expression of GITR in NK cells derived from breast cancer patients tended to be associated with the activation marker CD69. **(i)** No correlation of GITR and CD25 expression in T cells of HDs was observed. **(j)** In breast cancer patients GITR and CD25 expression was positively correlated. **(k-l)** In both, HDs and breast cancer patients the GITR expression on T cells correlated positively with T cell activation (CD69 expression). Since GITR expression seems to be regulated during immune cell activation, these data provides further evidence for the involvement of GITRL/GITR signaling in immune regulation in cancer.

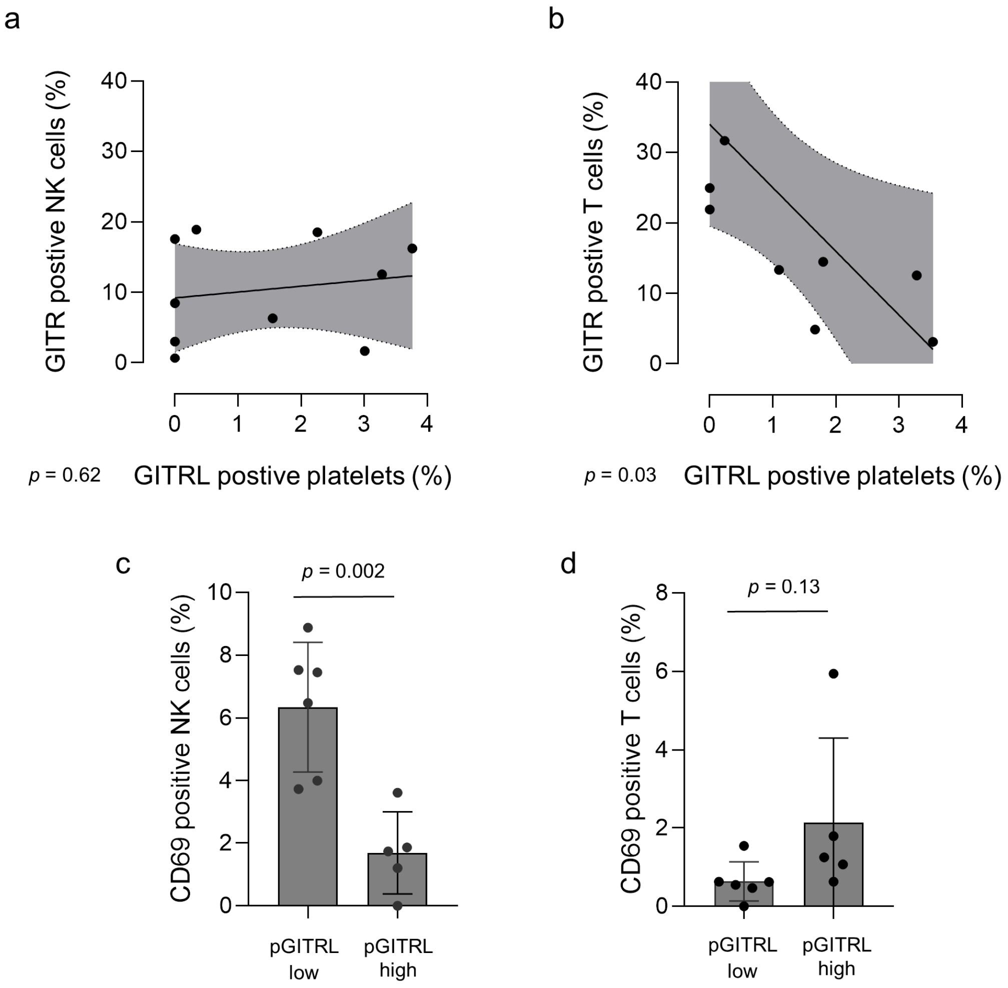

Suppl. Figure 3: Association of GITRL on platelets and corresponding immune cell activation and GITR expression. (a) The expression of GITR on the surface of NK cells is not associated with platelet-expressed GITRL (pGITRL) levels in breast cancer patients. **(b)** The expression of GITR on the surface of T cells is negatively associated with pGITRL levels in breast cancer patients (n=10). This suggests a downregulation of the T cell activating receptor GITR upon interaction with pGITRL which may serve as feedback loop to regulate GITRL/GITRL-mediated T cell activation. **(c)** Patients showing low levels of pGITRL displayed higher level of activated (CD69 positive) NK cells compared to those with high levels of pGITRL. **(d)** Patients with high levels of pGITRL tended to higher CD69 expression in T cells. This data might reflect the immune regulative function of pGITRL in breast cancer. The median of the respective cohort was used to define "*high*" and "*low*".

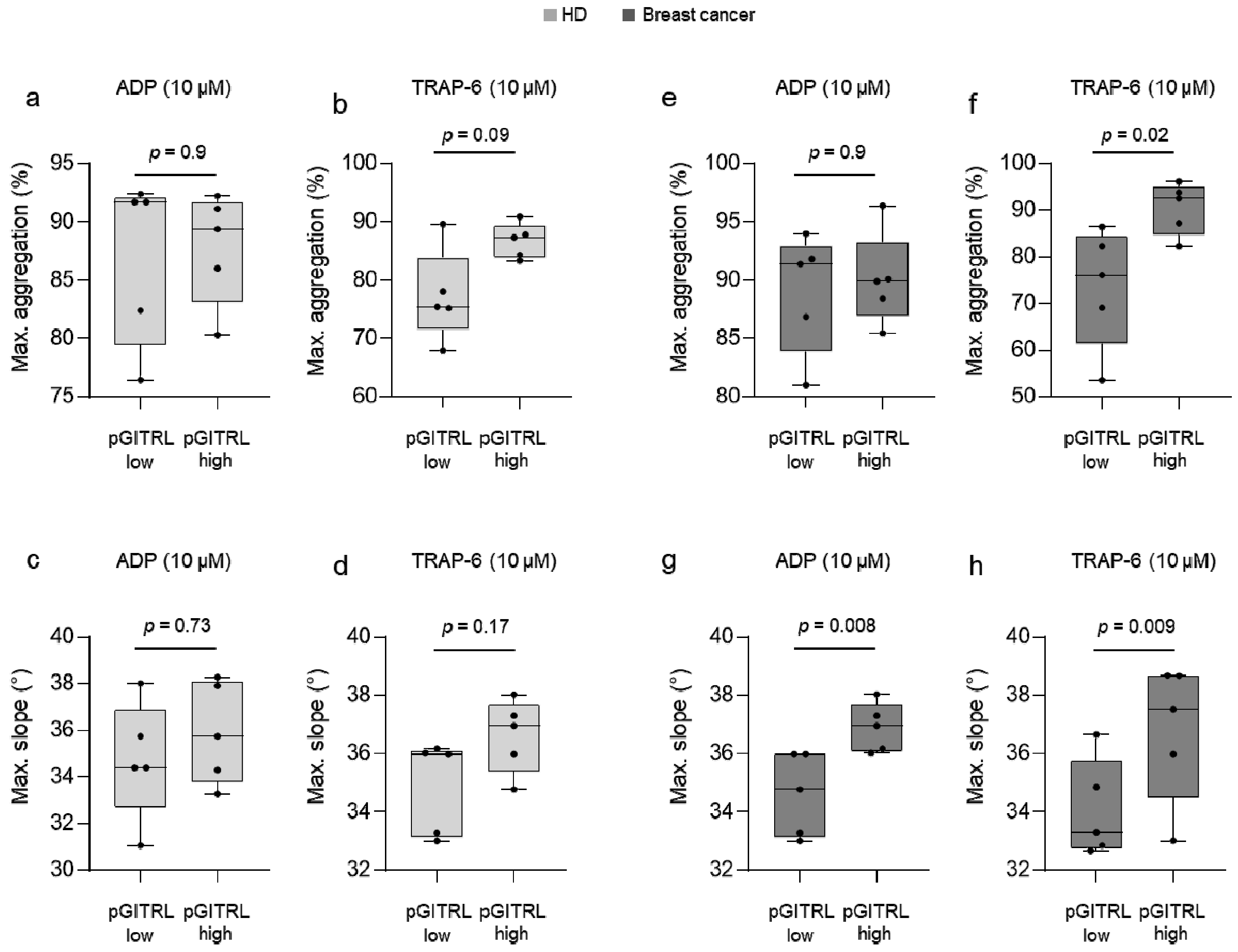

Suppl. Figure 4: Association of pGITRL and platelet aggregation. Platelet aggregation was induced using two classical platelet activators ADP or TRAP-6. **(a-b)** In HDs higher pGITRL level tended to be associated with higher maximal aggregation upon stimulation with 10 µM TRAP-6. ADP had no influence. **(c-d)** In line, after activation with TRAP-6 but not with ADP, HDs with high pGITRL expression tended to a higher maximal slope. **(e-f)** In breast cancer, patients with high level of pGITRL showed a higher max. aggregation upon TRAP-6 stimulation. ADP stimulation showed no differences in the pGITRL high and low group. **(g-h)** Interestingly, upon TRAP-6 and ADP stimulation the maximal slope was significantly higher in patients with high pGITRL levels. Considering the small number of patients (n=10), one could speculate that platelets containing higher levels of GITRL might be more sensitive. The median of the respective cohort was used to define "*high*" and "*low*".

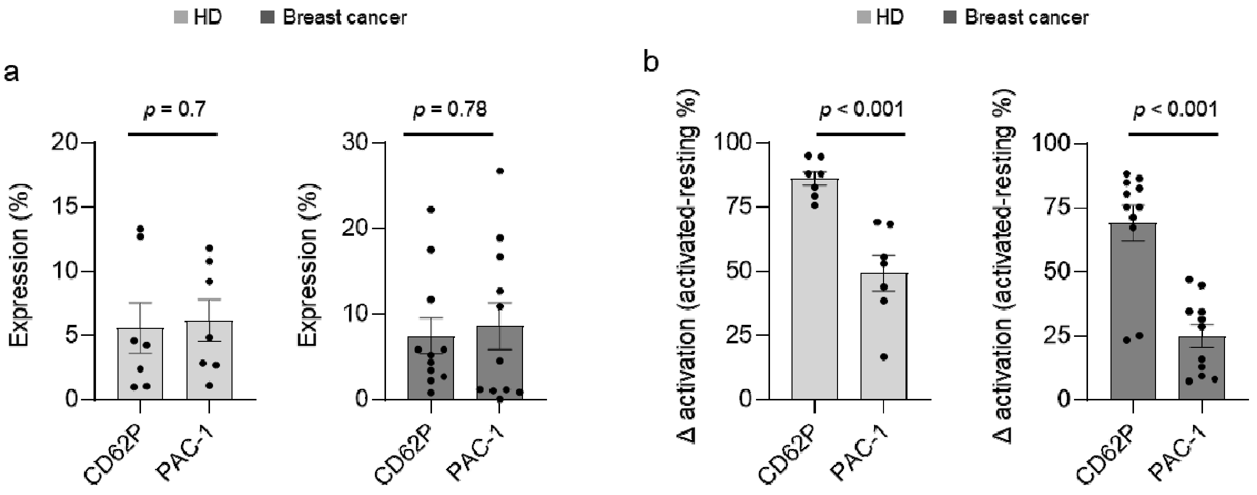

Suppl. Figure 5: Comparative analysis of the platelet activation markers CD62P and PAC-1. In order to cross-validate the platelet activation marker PAC-1 we analyzed the expression of PAC-1 and CD62P in 7 HDs and 10 breast cancer patients. **(a)** In our cohort of HDs and breast cancer patients expression level of PAC-1 and CD62P showed no significant differences. **(b)** Upon stimulation with 10 µM TRAP-6, the Δ activation (activated-resting in %) in the samples analyzed via CD62P was significantly higher compared to the Δ activation in the samples analyzed via PAC-1.
